# The Clinical Effectiveness of a Physiotherapy Delivered Physical and Psychological Group Intervention for Older Adults With Neurogenic Claudication: The BOOST Randomized Controlled Trial

**DOI:** 10.1093/gerona/glac063

**Published:** 2022-03-12

**Authors:** Esther Williamson, Graham Boniface, Ioana R Marian, Susan J Dutton, Angela Garrett, Alana Morris, Zara Hansen, Lesley Ward, Philippa J A Nicolson, David Rogers, Karen L Barker, Jeremy C Fairbank, Judith Fitch, David P French, Christine Comer, Christian D Mallen, Sarah E Lamb, BOOST Research Group, Mandy Maredza, Stavros Petrou, Julie Bruce, Frances Griffith, Gary Collins, Charles Hutchinson, Richard Gagen, Mandy Slack, Oliver Conway, Judith Fitch, Eileen Turner, John Arden, David Torgerson, Catherine Sackley, Candy McCabe, Stephanie Taylor, Catherine Hewitt, Anne Forster, Lindsey Bearne, Jim Watson

**Affiliations:** Nuffield Department of Rheumatology, Orthopaedics and Musculoskeletal Sciences, University of Oxford, Oxford, UK; College of Medicine and Health, University of Exeter, Exeter, UK; Nuffield Department of Rheumatology, Orthopaedics and Musculoskeletal Sciences, University of Oxford, Oxford, UK; Oxford Clinical Trials Research Unit, Centre for Statistics in Medicine, Nuffield Department of Orthopaedics, Rheumatology and Musculoskeletal Sciences, University of Oxford, Oxford, UK; Oxford Clinical Trials Research Unit, Centre for Statistics in Medicine, Nuffield Department of Orthopaedics, Rheumatology and Musculoskeletal Sciences, University of Oxford, Oxford, UK; Nuffield Department of Rheumatology, Orthopaedics and Musculoskeletal Sciences, University of Oxford, Oxford, UK; Nuffield Department of Rheumatology, Orthopaedics and Musculoskeletal Sciences, University of Oxford, Oxford, UK; Nuffield Department of Rheumatology, Orthopaedics and Musculoskeletal Sciences, University of Oxford, Oxford, UK; Department of Sport, Exercise and Rehabilitation, Northumbria University, Newcastle, UK; Nuffield Department of Rheumatology, Orthopaedics and Musculoskeletal Sciences, University of Oxford, Oxford, UK; Royal Orthopaedic Hospital NHS Foundation Trust, Birmingham, UK; Nuffield Department of Rheumatology, Orthopaedics and Musculoskeletal Sciences, University of Oxford, Oxford, UK; Oxford University Hospitals NHS Trust, Oxford, UK; Nuffield Department of Rheumatology, Orthopaedics and Musculoskeletal Sciences, University of Oxford, Oxford, UK; Patient and Public Involvement Representative; Manchester Centre for Health Psychology, University of Manchester, Manchester, UK; Physiotherapy Department, University of Leeds, Leeds, UK; Leeds Community Healthcare NHS Trust, Otley, UK; Primary Care Centre Versus Arthritis, School of Medicine, Keele University, Keele, UK; College of Medicine and Health, University of Exeter, Exeter, UK

**Keywords:** Exercise, Pain, Psychosocial, Rehabilitation, Spinal stenosis

## Abstract

**Background:**

Neurogenic claudication (NC) is a debilitating spinal condition affecting older adults’ mobility and quality of life.

**Methods:**

A randomized controlled trial of 438 participants evaluated the effectiveness of a physical and psychological group intervention (BOOST program) compared to physiotherapy assessment and tailored advice (best practice advice [BPA]) for older adults with NC. Participants were identified from spinal clinics (community and secondary care) and general practice records and randomized 2:1 to the BOOST program or BPA. The primary outcome was the Oswestry Disability Index (ODI) at 12 months. Data were also collected at 6 months. Other outcomes included ODI walking item, 6-minute walk test (6MWT), and falls. The primary analysis was intention-to-treat.

**Results:**

The average age of participants was 74.9 years (standard deviation [*SD*] 6.0) and 57% (246/435) were female. There was no significant difference in ODI scores between treatment groups at 12 months (adjusted mean difference [MD]: −1.4 [95% confidence intervals (CI) −4.03, 1.17]), but, at 6 months, ODI scores favored the BOOST program (adjusted MD: −3.7 [95% CI −6.27, −1.06]). At 12 months, the BOOST program resulted in greater improvements in walking capacity (6MWT MD: 21.7m [95% CI 5.96, 37.38]) and ODI walking item (MD: −0.2 [95% CI −0.45, −0.01]) and reduced falls risk (odds ratio: 0.6 [95% CI 0.40, 0.98]) compared to BPA. No serious adverse events were related to either treatment.

**Conclusions:**

The BOOST program substantially improved mobility for older adults with NC. Future iterations of the program will consider ways to improve long-term pain-related disability.

**Clinical Trials Registration Number:** ISRCTN12698674

Neurogenic claudication (NC) is a common, debilitating spinal condition affecting older adults (1). It presents as pain, discomfort, or other symptoms radiating from the spine into the buttocks and legs (2). Back pain is often present. Approximately 11% of community-dwelling older adults report symptoms consistent with NC (3,4). Symptoms are thought to arise from pressure on nerves and blood vessels in the spinal canal caused by degenerative narrowing of the spinal canal. The impact of narrowing is exacerbated by spinal position especially extension, and symptoms are provoked by walking or standing and relieved by sitting or lumbar flexion (2). Narrowing may or may not be evident on imaging (1), and if present, the condition is termed lumbar spinal stenosis. NC substantially affects an individual’s confidence and ability to walk and is associated with adverse health outcomes and reduced quality of life (3,5).

Despite the recognized severity of NC and lumbar spinal stenosis, there are insufficient numbers of high quality randomized controlled trials to inform clinical guidelines about the benefits of conservative interventions. In the absence of research evidence, 2 recent guidelines concluded that exercise/physical therapy might be considered despite the effects on neurogenic pain not being known (6,7). This lack of data extends to adverse outcomes of falls and muscle weakness. Behavioral interventions, including cognitive behavioral therapy have proven effective in managing nonspecific low back pain and promoting physical activity (8) but have not been investigated in NC. Hence, the aim of the Better Outcomes for Older People with Spinal Trouble (BOOST) Trial was to estimate the clinical effectiveness of a physiotherapist delivered physical and psychological intervention for older adults with NC compared to best practice advice.

## Method

### Design

This study was a pragmatic, multicentre, and randomized controlled superiority trial (RCT). The protocol, prespecified statistical analysis plan, and detailed description of the interventions are published elsewhere (9–11).

### Participants

Community-dwelling adults aged 65 years and older, who reported symptoms consistent with NC were eligible. Symptoms included a report of back pain and/or pain or other symptoms such as tingling, numbness, or heaviness that traveled from their back into their buttocks or legs in the last 6 weeks. Standing or walking made symptoms in the buttocks or legs worse and/or sitting or bending forward relieved these symptoms. Exclusion criteria included nursing home residents, inability to walk 3 meters independently, awaiting surgery, cauda equina syndrome or signs of serious pathology, cognitive impairment, and registered blind or unable to follow instructions in a group setting.

Potential participants were identified through community-based physiotherapy clinics and secondary care spinal clinics in 15 National Health Service (NHS) Trusts in England. Participants were also identified through a survey of general practices (The Oxford Pain, Activity and Lifestyle Survey [OPAL] cohort study) (12).

Once identified, potential participants were telephoned by a trained researcher (physiotherapist or nurse) for initial screening. If eligible and willing, potential participants attended an appointment to undergo an eligibility assessment conducted by the researcher. This included checking symptoms were consistent with NC and screening for cognitive impairment (defined as Abbreviated Mental Test score of 6 or less) (13) and serious pathology. All participants provided written informed consent prior to enrollment in the trial. Baseline data was then collected.

### Randomization and Masking

We used a secure web-based service provided by the Oxford Clinical Trials Research Unit. Randomization was stratified by recruitment center, age (65–74 years and <75 years), and gender, using variable, randomly selected block sizes of 3 and 6. Participants were randomized in a 2:1 ratio (intervention:control) to ensure that we could fill BOOST groups without participants experiencing long waiting times.

It was not possible to mask participants, physiotherapists delivering interventions or researchers assessing intervention fidelity. Participants were informed of their allocation at the first treatment session with the treating physiotherapist. Outcome assessors were masked to treatment allocation. During the conduct of the trial, the statistician had access to unmasked baseline summary data where required by the Data Monitoring Committee. The rest of the trial management team, including staff involved in data management, were masked to treatment allocation. Data cleaning and preparation of analysis code were undertaken by a masked statistician, and only once the data were formally locked, was the final analysis code run and allocation revealed.

### The BOOST Program

The experimental intervention was a combined physical and psychological group program (BOOST program) delivered by a physiotherapist in twelve 90-minute group sessions over 12-weeks (11). Participants were asked to undertake a home exercise program twice-weekly during and beyond the formal program.

First, each participant had an individual physiotherapy assessment. This included assessment of presenting NC symptoms, general health status, and current activity levels, including walking ability and screening for serious pathology. Physiotherapists assessed the participants’ ability to undertake the exercises to be completed during the group sessions and set the starting point for the exercises (sets, repetitions, and load) and walking program. This allowed individual tailoring. Four exercises targeted muscle strength (sitting knee extension, sit to stand, standing hip abduction, and standing hip extension). We used the Borg Rating Scale of Perceived Exertion for strength training to guide exercise prescription with the aim of achieving an adequate stimulus to promote strength gains. Participants were encouraged them to work at level 5–6/10 on this scale (the exercise feels hard) (14). Exercises also targeted balance and flexibility (hip flexor and calf stretch) while the walking circuit aimed to increase walking self-efficacy, dynamic balance, and mobility.

Participants attended the supervised sessions twice a week for sessions 1–6, weekly for sessions 7–9, and fortnightly for sessions 10–12. The twice-weekly home exercises were introduced during session 5, enabling participants to undertake the exercises with support before continuing independently. One and 2 months after completing the supervised sessions, physiotherapists conducted telephone reviews to promote adherence with the home exercises. The telephone calls followed a checklist and identified barriers to independent exercises, facilitated problem solving and allowed the physiotherapist to provide additional tailoring of the program as necessary.

Each group session followed the same format. The first 30 minutes was education and discussion based on a cognitive behavioral approach (CBA) to encourage adherence with the program. This was followed by the exercise element which took approximately 1 hour. There was a short warm-up of seated exercises (arm raises, trunk rotation, pelvic tilting, and knee lifts). Then participants completed their individually tailored strength, balance, and flexibility exercises which were progressed over the 12-weeks. The strengthening exercises were progressed by increasing the number of sets and repetitions, adding/increasing load, or adding speed. These exercises were also the home exercises. Participants then undertook a 20-minute supervised walking circuit which was progressed by increasing the distance/time walked, increasing walking speed, and adding challenges such as obstacles (stairs or walking outside) or adding weights. Participants were guided to gradually increase their walking distance during their home exercise program.

### The Control Intervention

The control intervention was best practice advice (BPA) delivered during individual physiotherapy appointments. The first appointment (60 minutes) included an assessment to tailor the advice and education provided. The assessment covered presenting NC symptoms, general health status and current activity levels, screening for serious pathology, spinal range of movement, and walking ability. Verbal and written advice and education were provided including education about NC, being physically active, use of medications, when to seek more advice and prescription of up to 4 home exercises. Flexion and trunk stabilization were recommended but other exercises were allowed based on the assessment. If indicated, a walking aid was prescribed. Ideally, the control intervention was delivered in 1 session. If the physiotherapist felt it was necessary, then up to 2 review appointments were permitted (30 minutes each) to re-enforce advice and review exercises or walking aids. Physiotherapists could not provide treatments such as manual therapy, acupuncture, or supervised exercise sessions.

All physiotherapists attended training in intervention delivery and trial procedures. Physiotherapists completed 2–3 hours of online training prior to attending a BOOST program training day (7 hours). BPA training was delivered in 2–3 hours on a separate day. Physiotherapists completed a treatment log for each participant. The research team observed the intervention sessions to monitor intervention delivery. A structured checklist was used to assess the delivery of the core elements of interventions (Supplementary Table S1) which was scored as not completed, partially completed, or fully completed. Initial observations were used to provide feedback and support physiotherapists to deliver the interventions. Later in the trial, these visits were fidelity assessments to understand how the intervention would be implemented in a real-world clinical setting with no feedback to the physiotherapists.

### Data Collection

Participants completed a questionnaire, and a masked researcher conducted physical testing at baseline, 6, and 12 months after randomization. If participants did not attend the follow-up appointment, then the physical tests were not completed and participants were sent a postal questionnaire. If the questionnaire was not returned after 2 reminders, then the study team collected core outcomes over the telephone, where possible.

### Baseline Variables

Descriptive baseline data included demographic data, weight and height, self-reported comorbidities (based on (15), with multimorbidity defined as 2 or more health conditions (16)), other pain problems measured using the Nordic Pain Questionnaire (17), use of walking aids inside, self-rated walking speed (18), and change in mobility in the last year.

The STarT Back Screening Questionnaire was completed, and participants categorized according to their risk (low, medium, or high) of developing persistent, disabling symptoms (19). Baseline psychological factors included confidence to exercise (Exercise Self-efficacy Scale [short version]) (20), confidence to manage their leg and back symptoms, intentions to carry out home exercises, walking self-efficacy (21), and fear-avoidance (Fear Avoidance Beliefs Questionnaire) (22). The Attitude to Aging Questionnaire (physical changes subscale) was completed (23).

### Outcome Measures

#### Primary outcome

The primary outcome was the Oswestry Disability Index (ODI v2.1a, https://eprovide.mapi-trust.org/instruments/oswestry-disability-index) at 12 months after randomization. This participant reported measure of pain-related disability is scored 0–100 with a higher score indicating greater disability.

#### Secondary outcomes

Participants underwent physical testing including the 6 minute walk test (6MWT), Short Physical Performance Battery (SPPB, range 0–12, higher score indicates better physical performance) (24), and a measure of hand grip strength (25).

Patient reported walking disability was measured using the ODI walking item (range 0–5, higher score indicating greater disability). Physical activity was measured using 2 items from the Rapid Assessment Disuse Index (time moving on feet, time spent sitting; range 1–5, lower score indicates greater duration moving/sitting) (26).

Participants reported falls and related injuries were collected by recall over a 6 month period using methods recommended by the Prevention of Falls Network Europe (ProFANE) (27). Frailty was measured using the Tilburg Frailty Indicator (TFI; range 0–15, higher score indicates greater frailty, physical subscale: range 0–8; psychological subscale: range 0–4) (28).

Participants reported outcomes relating to symptoms were measured using the Swiss Spinal Stenosis Questionnaire (SSSQ) symptom subscale (range 1–5, higher score indicates greater symptom severity) (29), pain troublesomeness scale (range 0–5, higher score indicates greater troublesomeness) (30), and global rating of change (range 0–6, lower score indicates improvement) (31). Satisfaction with changes in back and leg pain and satisfaction with treatment was measured using a 5-point scale constructed for the trial (range 0–4, higher score indicates greater satisfaction).

We collected adherence to home exercises via self-reported exercise frequency at follow-up and adverse events related to the interventions (Supplementary Materials for more information).

### Sample Size

At 80% power and 5% 2-sided significance levels, a sample size of 321 participants (214 in the intervention group and 107 in the BPA group) was required. With an inflation for potential loss to follow-up (20%) this led to an overall target of 402 (268 intervention, 134 control). The sample size assumed a between-group difference of 5 points in the ODI to be clinically significant, with a baseline standard deviation (*SD*) of 15 (32).

### Statistical Analysis

The primary outcome of ODI at 12 months follow-up was analyzed in an intention-to-treat (ITT) population and effect estimates with their 95% confidence intervals (CI) were reported at a 0.05 significance level. The ODI difference between the 2 treatment groups was estimated using a repeated measures linear mixed effects regression multilevel model with fixed effects for participant age, gender, and baseline ODI, and random effects for recruiting center and observations within-participant (6 and 12 months). To allow the treatment effect estimation at each follow-up time point, a treatment-by-time point interaction was also included in the model, with time point treated as categorial. Missing items within scales were dealt with based on published instrument recommendations. All participants with baseline and at least 1 follow-up outcome value were included in the likelihood-based estimation of the mixed effects model in the analysis, under the missing at random assumption.

A model additionally accounting for potential heterogeneity due to the treating physiotherapist was assessed in a sensitivity analysis. As multiple physiotherapists delivered some BOOST groups, the physiotherapist delivering the highest number of sessions was selected for the model. Similarly, we assessed if there was a group effect by including the BOOST group attended by each participant in a separate model. The robustness of the primary analysis for the primary outcome among participants compliant with treatment was conducted using a complier average causal effect (CACE) analysis (33). Compliance with the BOOST program was defined as attending at least 9 out of the 12 sessions (75%).

Secondary outcomes were analyzed in the ITT population, using similar model specifications for linear, logistic or, ordinal logistic mixed effects regression models as appropriate and adjusting for the relevant baseline covariate where applicable. Analyses of secondary outcomes were considered supportive of the primary outcome analysis. All analyses were carried out using Stata version 15.1 (StataCorp, College Station, TX).

### Ethical Approval

Ethics approval for the BOOST trial was given by the London-Brent National Research Ethics Committee (REC number 16/LO/0349) on March 3, 2016.

## Results

**Figure 1.**
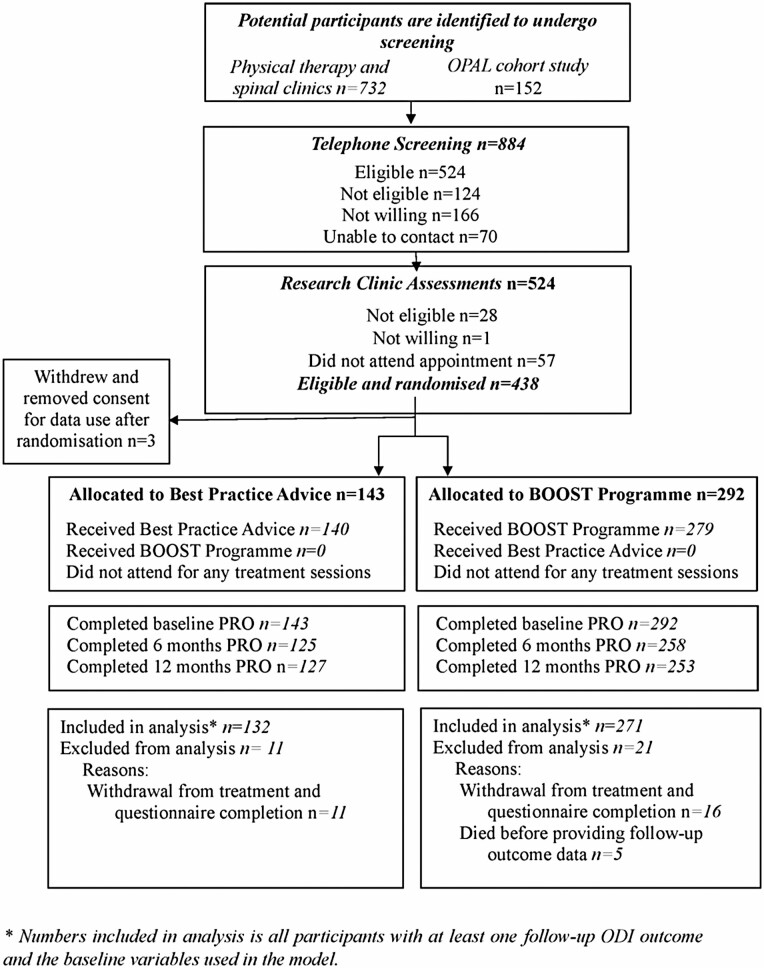
Consort diagram.

Participant flow is shown in Figure 1. Participants were recruited between August 1, 2016 and August 29, 2018 at 15 trial sites. Clinical staff identified 732 potential participants to undergo screening by researchers. From the OPAL cohort, we identified 152 potential participants. After screening, a total of 438 participants were eligible and willing to participate, provided informed consent and were randomized. Three participants withdrew after randomization and removed consent data use (all allocated to BOOST program, 2 withdrew before their first physiotherapy appointment, 1 withdrew after their first appointment). Therefore, 435 participants (BPA *n* = 143, BOOST program *n* = 292) were included in the trial.

The primary outcome was obtained for 88.0% (383/435) and 87.4% (380/435) of participants at 6 months and 12 months, respectively with 93.0% (403/435) contributing data to the primary analysis. During the follow up period, 6.2% (27/435) withdrew. The most common reason for withdrawal was health issues unrelated to their NC or the trial. There was no evidence of a differential loss to follow-up between the 2 groups. All reported deaths were found to be unrelated to the intervention.

### Baseline Characteristics

Participants had a mean age of 74.9 years (*SD* 6.0) and were predominantly white (91.9% [400/435]). The randomized groups were well-matched on baseline characteristics (Tables 1 and 2). In the BPA group, a larger proportion of participants were classified as frail (55.9% vs 44.5%) according to the Tilburg Frailty Index but other markers of frailty (6MWT, SPPB, and hand grip strength) were similar. Eighty-one percent (351/435) had multimorbidity. The most commonly reported conditions were arthritis (272/435; 62.5%), high blood pressure (252/435; 57.9%), angina/heart problems (104/435; 23.9%), digestive problems (87/435; 20.0%), and diabetes (73/435; 16.8%).

**Table 1.** Baseline Characteristics (Mean [Standard Deviation] or *n* [%; Unless Stated])

Variables*	BPA (*n* = 143)	BOOST Program (*n* = 292)	Overall (*n* = 435)
Age (years) at baseline	75.0 (5.6)	74.8 (6.2)	74.9 (6.0)
Female	83 (58.0%)	163 (55.8%)	246 (56.6%)
White ethnicity	132 (92.3%)	268 (91.8%)	400 (91.9%)
Relationship status			291 (66.9%)
Married/civil union/cohabiting	97 (67.8%)	194 (66.40%)	
Unmarried/separated/divorced	16 (11.2%)	31 (10.7%)	47 (10.8%)
Widow/widower	30 (21.0%)	67 (22.9%)	97 (22.3%)
Care requirements			
Has an unpaid carer	31 (21.7%)	54 (18.5%)	85 (19.5%)
Has a paid carer	6 (4.2%)	10 (3.4%)	16 (3.7%)
Work status			
Retired	125 (87.4%)	263 (90.1%)	388 (89.2%)
Working (full or part-time)	10 (6.9%)	24 (8.2%)	34 (7.8%)
Education			
None or primary education	4 (2.8%)	18 (6.2%)	22 (5.1%)
Secondary education	80 (55.9%)	170 (58.2%)	250 (57.5%)
Higher professional/university education	59 (41.3%)	104 (35.6%)	163 (37.5%)
Smoking status			
Never smoked	61 (42.7%)	136 (46.6%)	197 (45.3%)
Former smoker	75 (52.4%)	140 (47.9%)	215 (49.4%)
Current smoker	7 (4.9%)	16 (5.5%)	23 (5.3%)
Body mass index	30.0 (5.4)	29.9 (4.8)	29.9 (5.0)
Number of comorbidities reported, median (IQR)	3 (2, 4)	2 (2, 4)	2 (2, 4)
Nordic Pain Questionnaire			
Single-site pain	14 (9.8%)	16 (5.5%)	30 (6.9%)
Multisite pain	129 (90.2%)	276 (94.5%)	405 (93.1%)
STarTBack			
Low risk	48 (33.8%)	109 (37.6%)	157 (36.3%)
Medium risk	67 (47.2%)	138 (47.6%)	205(47.5%)
High risk	27 (19.0%)	43 (14.8%)	70 (16.2%)
Classified as frail,^†^*n* (%)	80 (55.9%)	130 (44.5%)	210 (48.3%)
Self-rated outdoor walking speed, median (IQR)	4 (3, 4)	4 (3, 4)	4 (3, 4)
Change in mobility			
Better than one year ago	9 (6.3%)	15 (5.2%)	24 (5.5%)
About the same	30 (21.0%)	86 (29.5%)	116 (26.7%)
Worse than one year ago	104 (72.7%)	191 (65.4.8%)	295 (67.8%)
Use of walking aids outside			
Yes	40 (28.0%)	75 (25.7%)	115 (26.4%)
Sometimes	28 (19.6%)	55 (18.8%)	83 (19.1%)
Use of walking aids inside			
Yes	9 (6.3%)	16 (5.5%)	25 (5.7%)
Sometimes	15 (10.5%)	35 (12.0%)	50 (11.5%)
Attitudes to aging questionnaire^‡^	28.7 (6.6)	29.0 (5.9)	28.9 (6.1)
Intention to exercise, median (IQR)^§^	6 (6, 7)	6 (6, 7)	6 (6, 7)
Exercise self-efficacy scale, median (IQR)^‖^	68 (54, 80)	70 (52, 81)	69 (53, 80)
Walking self-efficacy^¶^	5.3 (3.3)	5.7 (3.3)	5.6 (3.3)
Confidence in ability to self-manage symptoms^#^	6.1 (1.78)	6.1 (1.81)	6.1 (1.80)
Fear-avoidance beliefs^**^	12.7 (5.4)	13.0 (6.1)	12.9 (5.9)

*Note:* IQR = interquartile range.

*Baseline data for clinical outcomes is available in Tables 2 and 3.

^†^Based on the Tilburg Frailty Index score of ≥5.

^‡^Range 8–40, higher score indicates a more positive attitude to aging.

^§^Range 1–7, higher scores indicates stronger intensions.

^‖^Range 0–90, higher score indicates greater self-efficacy.

^¶^Range 0–10, higher score indicates greater self-efficacy.

^#^Range 0–10 indicates greater self-efficacy to walk half a mile.

^**^Range 4–24, higher scores indicating greater fear avoidance.

**Table 2.** Patient Reported Outcomes

Outcome	Best Practice Advice			BOOST Program		Between-Group Difference* (95% CI)	*p* Value
		*n*	Unadjusted Mean (*SD*)*	*n*	Unadjusted Mean (*SD*)*		
ODI*	Baseline	143	32.3 (14.2)	292	33.2 (13.7)	n/a	
	6 months	125	33.2 (15.9)	258	30.2 (16.5)	−3.7 (−6.27, −1.06)	**.006**
	12 months	127	33.0 (17.4)	253	31.7 (18)	−1.4 (−4.03, 1.17)	0.281
ODI Walking Item*	Baseline	143	1.8 (1.2)	292	1.8 (1.2)	n/a	
	6 months	125	1.8 (1.3)	258	1.6 (1.3)	−0.2 (−0.44, −0.02)	**.033**
	12 months	126	1.9 (1.4)	253	1.6 (1.4)	−0.2 (−0.45, −0.01)	**.041**
RADI―hours moving,^†^ median (IQR)	Baseline	143	3.0 (3.0, 4.0)	292	3.0 (2.0, 4.0)	n/a	
	6 months	125	3.0 (3.0, 4.0)	256	3.0 (2.0, 4.0)	0.6 (0.39, 0.87)^‡^	**.008**
	12 months	127	3.0 (2.0, 4.0)	248	3.0 (2.0, 4.0)	0.9 (0.61, 1.35)^‡^	.633
RADI―hours sitting,^†^ median (IQR)	Baseline	143	3.0 (2.0, 3.0)	292	2.0 (2.0, 3.0)	n/a	
	6 months	125	3.0 (2.0, 3.0)	256	2.0 (2.0, 3.0)	0.8 (0.49, 1.14)^‡^	.174
	12 months	127	2.0 (2.0, 3.0)	250	2.0 (2.0, 3.0)	1.0 (0.68, 1.55)^‡^	.886
TFI^§^	Baseline	143	4.9 (2.50)	286	4.4 (2.70)	n/a	
	6 months	124	5.2 (2.70)	246	4.4 (2.80)	−0.4 (−0.80, 0.05)	.085
	12 months	124	5.2 (2.80)	241	4.8 (3.00)	0.1 (−0.34, 0.52)	.676
TFI―physical subscale^§^	Baseline	143	3.0 (1.60)	290	2.6 (1.70)	n/a	
	6 months	125	3.1 (1.80)	250	2.6 (1.80)	−0.3 (−0.61, 0.00)	**.052**
	12 months	125	3.1 (1.90)	245	2.8 (1.90)	0.0 (−0.33, 0.29)	.918
TFI―psychological subscale^§^	Baseline	143	1.1 (1.00)	292	1.0 (1.10)	n/a	
	6 months	125	1.2 (1.10)	256	1.0 (1.00)	−0.1 (−0.31, 0.05)	.152
	12 months	127	1.2 (1.00)	251	1.2 (1.10)	0.1 (−0.13, 0.24)	.563
One of more falls,^‖^*n* (%)	Baseline	143	50 (35%)	292	115 (39.4%)	n/a	
	Over 12 months	125	59 (41.3%)	257	96 (32.9%)	0.6 (0.40, 0.98)^‡^	**.041**
Broken bones following a fall,^¶^*n* (%)	Baseline	143	4 (2.8%)	292	8 (2.7%)	n/a	
	Over 12 months	127	9 (7.1%)	253	17 (6.7%)	n/a	
SSSQ symptom subscale^§^	Baseline	143	3.0 (0.60)	292	3.0 (0.60)	n/a	
	6 months	119	2.8 (0.80)	247	2.7 (0.80)	−0.2 (−0.28, −0.02)	**.025**
	12 months	113	2.8 (0.80)	229	2.7 (0.80)	−0.1 (−0.19, 0.08)	.428
Troublesomeness,^†^ median (IQR)	Baseline	125	4.0 (3.0, 4.0)	258	4.0 (3.0, 4.0)	n/a	
	6 months	125	3.0 (3.0, 4.0)	258	3.0 (2.0, 4.0)	0.5 (0.27, 0.87)^‡^	**.014**
	12 months	127	3.0 (2.0, 4.0)	253	3.0 (2.0, 4.0)	0.8 (0.45, 1.43)^‡^	.454
Global rating of perceived change^#^	Baseline		n/a		n/a	n/a	
	6 months	125	4.0 (3.0, 5.0)	257	3.0 (2.0, 5.0)	−0.4 (−0.75, −0.11)	**.009**
	12 months	127	4.0 (3.0, 5.0)	252	4.0 (3.0, 5.0)	0.0 (−0.30, 0.34)	.902
Satisfaction: treatment,^**^ median (IQR)	Baseline		n/a		n/a	n/a	
	6 months	125	3.0 (2.0, 4.0)	256	3.0 (2.0, 4.0)	2.5 (1.41, 4.44)^‡^	**.002**
	12 months	126	2.0 (2.0, 4.0)	248	3.0 (2.0, 4.0)	2.7 (1.54, 4.83)^‡^	**.001**
Satisfaction: change in back and leg problems,^**^ median (IQR)	Baseline		n/a		n/a	n/a	
	6 months	125	2.0 (2.0, 3.0)	256	3.0 (2.0, 4.0)	3.1 (1.63, 6.08)^‡^	**.001**
	12 months	126	2.0 (2.0, 3.0)	247	2.0 (2.0, 3.0)	1.8 (0.91, 3.38)^‡^	.095

*Notes:* *Unless indicated. CI = confidence interval; IQR = interquartile range; ODI = Oswestry Disability Index; RADI = Rapid Assessment Disuse Index; *SD* = standard deviation; SSSQ = Swiss Spinal Stenosis Questionnaire; TFI = Tilburg Frailty Index. Bold values are statistically significant findings.

*ODI analysis adjusted for age, gender, and baseline ODI. Model includes repeated measures with random effects for participant and center. Four hundred and three participants contributed to the model.

^†^Mixed effects ordinal logistic regression analysis adjusted for age, gender, and baseline score, with repeated measures within participant and center, and time point-by-treatment interaction.

^‡^Adjusted odds ratio (95% CI).

^§^Mixed effects linear regression analysis adjusted for age, gender, and baseline score, with repeated measures within participant and center, and time point-by-treatment interaction.

^‖^Mixed effects logistic regression analysis adjusted for age, gender, and baseline score, with repeated measures within participant and center, and time point-by-treatment interaction.

^¶^Given the low event rate reported for number of broken bones following fall, no statistical test was used for comparison.

^#^Mixed effects linear regression analysis adjusted for age and gender with repeated measures within participant and center, and time point-by-treatment interaction.

^**^Participant satisfaction mixed effects ordinal logistic regression analysis adjusted for age and gender with repeated observations within participant and center; breakdown of scores from 0 to 4 are presented in Supplementary Table S3.

### Intervention Delivery

Sixty-nine physiotherapists delivered the interventions. Thirty physiotherapists delivered BPA, 34 physiotherapists delivered the BOOST program, and 5 physiotherapists delivered both. In total, 24/143 (16.8%) participants allocated to BPA were treated by physiotherapists who were also trained in the BOOST intervention.

Of the 143 participants allocated to BPA, 140 (98%) received the intervention. The mean time from randomization to the first BPA appointment was 34.7 (*SD* 20.8) days. Most commonly, participants attended 2 BPA appointments (41.3% [59/143]). The reasons that 3 participants did not attend any appointments were health problems, family concerns, and a decision to have spinal surgery.

Of the 292 participants allocated to the BOOST program, 279 (96.0%) attended the individual physiotherapy assessment (mean time from randomization to appointment: 31.2 [*SD* 27.3] days). Thirteen participants (4.5%) did not attend this assessment. Reasons for nonattendance included sickness, lack of time, travel distance, work commitments, group allocation, and considering surgery. After the individual assessment, participants joined the next available group (mean time from randomization to the first group session: 58.7 [*SD* 38.51] days). In total, 203/292 (69.5%) attended at least 9 of the 12 sessions indicating compliance. Having attended the individual assessment, 13 participants (4.5%) subsequently did not attend any group sessions. The most common reasons for group nonattendance were holidays or sickness.

We conducted 123 observations of treatment sessions including 48 fidelity assessments. Interventions were delivered to a high standard. Eighteen fidelity assessments were undertaken of BPA sessions and 97.2% of checklist items were fully achieved. Thirty fidelity assessments of the BOOST program group sessions were conducted with 97.4% of checklist items fully achieved. Monitoring of treatment logs showed that exercises were progressed regularly across the key parameters including increased repetitions and load, and addition of speed to the strengthening exercises. During the walking circuit, increasingly difficult elements were added to challenge balance such as increased speed, carrying weights, and negotiating obstacles.

### Primary Outcome

Participants randomized to BPA showed a small increase in ODI scores at 6 months with very little subsequent change at 12 months. BOOST program participants showed a reduction in ODI scores at 6 months which increased again at 12 months but remained lower than baseline scores. At the 12-month primary end-point, there was no statistically significant difference in ODI scores between the 2 treatment groups (adjusted mean difference −1.4, 95% CI −4.03 to 1.17). There was a statistically significant difference in ODI in favor of the BOOST Program group (adjusted mean difference −3.7, 95% CI −6.27 to −1.06) at 6 months. There was no evidence of a therapist or group effect.

In the CACE analysis, the difference favoring the BOOST program was larger, reaching the predefined clinically significant threshold (5 points on the ODI) when group attendance was taken into consideration (−5.0, 95% CI −8.02 to −1.88) at 6 months. At 12 months, this difference was reduced (−2.4, 95% CI −6.02 to 1.32). Among noncompliers there was a greater proportion characterized as frail (50.6% vs 41.9%), having fallen in the previous year (43.8% vs 37.4%), and reporting very/extremely troublesome back and leg pain (57.3% vs 51.2%) compared to compliers.

### Secondary outcomes (Tables 2 and 3)

**Figure 2.**
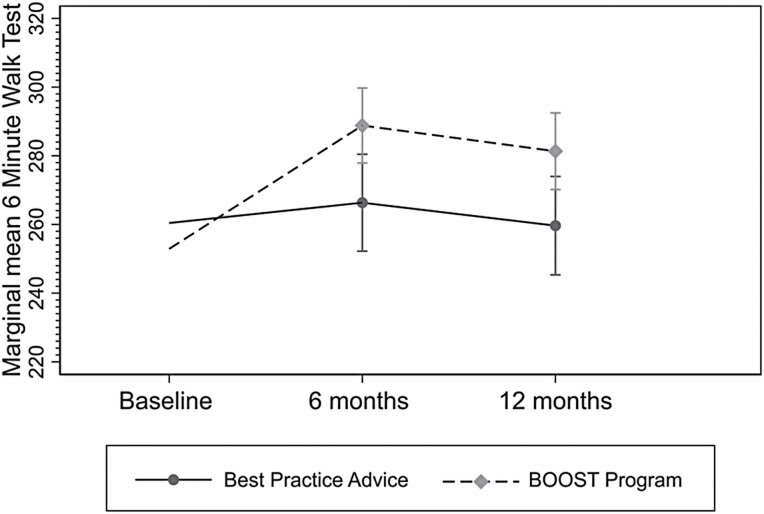
Marginal adjusted mean Six Minute Walk Test results from baseline to 12 months by treatment group.

The BOOST program had a lasting impact on walking capacity (6MWT; Figure 2) at 6 and 12 months follow up favoring the BOOST program. BPA participants showed very little change across the 2 follow-up time points. A similar response was observed for physical performance (SPPB). Changes in grip strength favored the BOOST program at 6 months but there was no between-group difference at 12 months.

**Table 3.** Outcomes―Physical Tests

Outcome		Best Practice Advice		BOOST Program		Between-Group Difference (95% CI)	*p* Value
		n	Unadjusted Mean (SD)*	n	Unadjusted Mean (SD)*		
Six minute walk test*	Baseline	143	260.4 (101.30)	292	252.9 (98.10)	n/a	
	6 months	118	266.3 (103.40)	240	283.5 (99.40)	22.5 (7.11, 37.82)	**.004**
	12 months	111	263.2 (106.70)	216	284.7 (105.40)	21.7 (5.96, 37.38)	**.007**
SPPB,* median (IQR)	Baseline	143	9.0 (8.00, 11.00)	291	9.0 (7.00, 11.00)	n/a	
	6 months	118	9.0 (7.00, 11.00)	245	10.0 (8.00, 11.00)	0.6 (0.19, 0.97)	**.003**
	12 months	112	9.5 (7.00, 11.00)	218	10.5 (8.00, 12.00)	0.4 (0.00, 0.80)	**.052**
Grip Strength*	Baseline	143	26.7 (10.50)	292	26.7 (10.50)		
	6 months	118	26.1 (11.10)	247	27.1 (10.60)	1.2 (0.28, 2.11)	**.010**
	12 months	112	26.4 (11.30)	225	27.0 (10.60)	0.9 (-0.08, 1.79)	.073

*Notes:* *Unless indicated. CI = confidence interval; IQR = interquartile range; *SD* = standard deviation; SPPB = short physical performance battery. Bold values are statistically significant findings.

*Mixed effects linear regression analysis adjusted for age, gender, and baseline score, with repeated measures within participant and center, and time point-by-treatment interaction.

The BOOST program reduced walking disability (ODI walking item) at 6 and 12 months compared to BPA. BOOST participants were more likely to spend more time on their feet at 6 months but not 12 months. There was no impact on time spent sitting.

BOOST program participants had a substantially reduced risk of reporting a fall over the 12-month period. The proportion of participants reporting a fracture following a fall was very small but similar between groups. Physical frailty scores favored the BOOST program (TFI physical subscale) at 6 months with BOOST participants demonstrating less decline than the BPA group. There was no difference at 12 months. There was no impact on overall TFI or psychological subscale.

Both groups reported a small reduction in SSSQ symptoms subscale scores at 6 months and these were larger for the BOOST program. Small reductions were maintained at 12 months and there was no longer a difference between the groups at 12 months. Similar findings were observed for troublesomeness, Global Rating of Change, and satisfaction with changes in back and leg problems. BOOST program participants were more likely to be satisfied with their treatment at 6 and 12 months compared to the control group.

### Exercise Adherence

Participants were asked how often they performed their home exercises. At 6 months, 190/257 (73.9%) BOOST program participants reported performing their exercises at least twice per week that reduced to 143/250 (57.2%) at 12 months. At 6 months, 102/125 (81.6%) BPA participants reported doing their exercises at least twice a week that reduced to 89/125 (71.2%) at 12 months.

### Adverse Events

One serious adverse event (cardiac symptoms) occurred during a BOOST group session which was deemed unrelated to the intervention. There were no serious adverse events reported for BPA. There were 12 adverse events reported for the BOOST Program (Supplementary Table S2). Four were assessed as definitely related to the program including aggravation of joint pains (*n* = 2), a fall during the walking circuit (no injuries), and skin irritation by an ankle weight. Two adverse events were reported for BPA and neither were definitely related to the treatment.

## Discussion

The BOOST program improved walking capacity and physical performance and reduced walking disability and falls risk compared to a control intervention of BPA for older adults with NC at 12 months follow up. There were also improvements in pain-related disability at 6 months favoring the BOOST program but only a small difference between groups was maintained at 12 months which was not statistically significant. Symptom reduction followed a similar pattern. There was very little change in the scores of BPA participants for outcomes generally over time.

The biggest impact was on mobility. Baseline walking distances were well below published values for healthy older people demonstrating the substantial impact that NC has on walking ability (34). The mean baseline 6MWT distances for BOOST participants were lower than other published baseline values of NC cohorts (eg, (35), baseline 6MWT 315m, mean age = 67 years) but BOOST participants were older. As people age, we expect a decline in walking over time rather than improvement (36), yet, participants attending the BOOST program demonstrated changes in walking capacity with observed improvements within the published values for clinically important differences for the 6MWT (37). These improvements were not observed in BPA participants who changed very little. Chronic pain, such as that experienced from NC, which is a chronic degenerative condition, is associated with falls in older people (38). The BOOST program reduced falls risk by approximately 40% over 12 months which is more effective than most community-based falls prevention programs (39). These lasting improvements in mobility and reduced falls risk are important outcomes for older adults. Active independence is one of the key concerns of older people, and maintaining mobility is integral to this (40). Qualitative research demonstrates a desire by older people to improve their walking even if they cannot alleviate the pain of NC (41). Despite the value of mobility to older people, its importance as an outcome in clinical trials of treatments of NC or spinal stenosis is often overlooked, especially in surgical trials. Two recent network meta-analyses of treatments for spinal stenosis evaluated effectiveness solely on pain and disability, failing to evaluate the impact on walking (42,43). An exception to this is a surgical trial currently being conducted which has chosen improvement in walking capacity as the coprimary outcome along with the ODI (44).

The short-term reduction in pain-related disability among BOOST participants compared to BPA suggests that while participants are engaged with the program it effectively reduces pain-related disability. The between-group difference increased when group attendance was taken into account. However, when the intervention stops, the impact on pain-related disability reduces. Although, participants were still capable of walking further (6MWT improvements were maintained), it no longer translates into reduced pain-related disability.

We noted a reduction in independent exercise in the BOOST Group between 6 and 12 months follow up which may explain why improvements were not maintained. This finding is not unique to the BOOST program. Devereux-Fitzgerald et al. (45) found supervision by a health professional increases the perceived value of physical activity interventions enhancing engagement but this reduces when supervision ceases. Attendance at a group is enjoyable and provides increased social connections, but solo activities such as independent exercise are often considered boring leading to lack of motivation (45). Self-reported adherence with the home exercises was better in the BPA group who were given a less intensive home exercise program (up to 4 spinal mobility and/or stability exercise). BOOST participants may have perceived their home exercise as too onerous, and consideration should be given as to whether the unsupervised element of the program can be optimized to maximize adherence. It may also be that participants experience a flare-up of their pain which is common in NC. We used a less intensive CBA than a previous trial evaluating a CBA (Back Skills Training Program (8)) which effectively reduced back pain-related disability long-term so this element of the BOOST program could be enhanced to assist participants to deal with increases in pain.

Three trials of note were recently published (46–49). Similar to the BOOST trial, all tested programs which included structured and progressive exercises to improve trunk and lower limb mobility, strength, and fitness. Participants also received manual therapy treatment to increase spinal movement. The Ammendolia program is most similar to the BOOST program including a CBA for pain management and structured walking program delivered over 12 sessions (47). It was compared to self-directed exercise (1 session). The Ammendolia program also resulted in lasting improvements in walking compared to the control providing further support for implementing these types of program. The 6-week (12 sessions) program evaluated by Minetama also included walking training which was done on a treadmill but did not address any psychological factors (48,49). It resulted in superior outcomes across multiple domains (walking, pain, and function) on completion of treatment compared to home exercises (48). Some benefits were retained at 12 months follow-up in regard to pain and function but unfortunately, they did not measure walking (49). The Schneider program did not have a focused walking element or use a CBA (46). This 3-arm study found no difference in walking between the 6-week experimental arm and control arm of medical care, suggesting one or both of these elements are important to achieve walking improvements.

Ensuring effective treatments are available to older people with NC is very important as, currently, treatment options are limited. There is little evidence supporting the use of medication (50). Careful consideration is needed before prescribing medication for older people due to potential side effects including falls (50). Surgery is an option with symptomatic spinal stenosis being the most common reason for spinal surgery in older adults (51). However, the effectiveness of surgery is unclear, and it exposes older people to considerable risk including wound infections, dural tears, and cardiorespiratory complications (52). Surgery is usually reserved for those who are fitter (and hence younger). Populations in surgical trials are considerably younger (42). Our participants had a mean age of 75 years, the majority were multimorbid and nearly half were frail. The BOOST program should be considered a worthwhile conservative treatment for older adults especially when they are not surgical candidates or face long waiting times for surgery due to the impact of the COVID-19 pandemic on NHS waiting lists.

We believe our trial to have considerable strengths. It was a pragmatic trial conducted across a range of NHS settings. We recruited participants from general practice, community-based physiotherapy clinics, and spinal clinics in secondary care hospitals lending to the generalizability of findings. Based on fidelity assessments, the BOOST program can be delivered to a high standard in different settings. The questions used to identify those with NC are commonly used in clinical practice and been shown to have high sensitivity and specificity to identify people with symptoms arising from spinal stenosis (1). This makes it easy for clinicians to identify people that would be suitable for the program without the need for MRI. The BOOST program was well-received by participants and despite the required commitment, the program was well-attended. However, compliance was lower amongst participants who were frail, reported falling, and had more troublesome symptoms. These individuals may require more support and encouragement to attend the program.

A limitation of the study is that 5 physiotherapists trained in delivery of the BOOST program also treated 24/143 participants (16.8%) allocated to BPA due to physiotherapist availability. However, the proportion of participants in the control arm exposed to potential contamination is well below the 30% threshold considered a serious threat (53). We carefully monitored intervention delivery using treatment logs and observation sessions to ensure the standardized protocols were followed. From fidelity assessments, we are confident that the risk of contamination between arms was minimized. We took all possible steps to mask the trial team, outcome assessors, and statisticians. It is possible that during the final analysis statisticians could deduce the allocation because of the unequal randomization, but at that stage the database was securely locked, and data could not be tampered with or changed.

There are some potential limitations related to the interventions. Firstly, we used the Borg Rating of Perceived Exertion to prescribe the BOOST program strengthening exercises. This is a pragmatic approach to exercise prescription that can be done easily in a clinical setting and is recommended as a suitable approach for prescribing resistance training for older adults (54). However, this approach may not be as accurate as using a method based on a percentage of 1 repetition maximum (%1RM) with the risk of under or overdosing. Finally, the participants attending the BOOST program had more contact time with the treating physiotherapist than those attending BPA. As this was a pragmatic trial, we did not account for this in our control intervention and using an attention control, such as that used by LaFave et al. (55), would have enabled us to disentangle the benefits of attention from the impact of the BOOST program.

The BOOST program could be optimized to maintain the impact on pain-related disability. In particular, strategies for improving long-term exercise adherence should be considered including additional support. Additional support could include booster sessions which has been shown to increase exercise adherence in populations with back pain and osteoarthritis (56). We will also consider enhancing the CB element to improve pain management. We plan to undertake further analysis of the BOOST data set to increase our understanding of participants’ response to the intervention and to understand the mechanisms of action including mediation analyses (57).

In conclusion, the BOOST program improves mobility and reduces falls for older adults with NC compared to BPA at 12 months. With limited treatment options available to older people with NC, implementation of the program should be considered. Future iterations of the program will consider ways to improve long-term pain-related disability.

## Supplementary Material

glac063_suppl_Supplementary_Material

## Clinical Collaborators

Birmingham Community NHS Trust: Lindsey Hurley (Principal Investigator [PI]), Claire Williams, Connor Ruffinato, Emma Komocki, Joe Golby, Jonathan Price, Lauren Shields, Lorraine Moylan, and Rowena Quirk. Cambridgeshire Community Services NHS Trust: Jenny Van Maurik (PI), Ben Whybrow, Greg Wisniewski, Mandy Whitlock, Matthew Pearson, Michelle Read, Ben Whybrow, Michelle Read, Oliver Richardson, and Rachel Renshaw. Croydon Health Services NHS Trust: Suzy Peerman (PI), Brinna Robertson-More, Emma Parker, Georgia Leher, Hilary Viggars, Lee McFarland, Louise Robles, and Lynette Boot. Dorset Healthcare University NHS Foundation Trust: Katrina Kennedy (PI), Christian Verrinder (PI), Brandon Bule, Chris Mott, Emma Bell, Josephine Moore, Kate Gough, Kathryn Francis, Louise Kazemi, Louise Pearce, and Susan Dowdle. East Cheshire NHS Trust: Simon Hogan (PI), Helen Ashton, Helen Lomas, Kerry Wallis, Laura Plant, Richard Saxton, and Vicki Austin. Gloucestershire Care Services NHS Trust: Caroline Hooper (PI), Emma Moule, Helen Fursman, Inge Carrington, Jo Godley, Karen McIlveen, and Sherry Claridge. Leeds Community NHS Healthcare Trust: Christine Comer (PI), John O’Hora, Sam Noble, Samantha Kelly, and Stephen Bell. Leeds Teaching Hospital NHS Trust: Denise Ross (PI), Joanne Draycott, and Tom Bindloss. Northwest Boroughs Partnership NHS Trust: Gill Dickson (PI), Aisling McGrath, Catherine Atherton, Catherine Quigley, Ceit Bannon, Emma Painter, Joanna Lunt, Matthew Martyniak, Paula Cleary, Peter Cuddy, and Rebecca McDonough. Oxford University Hospitals NHS Foundation Trust: Karen Barker (PI), Amanda Robertson, Bridget Gould, Claire Rogers, Colette Tracey, Helen Stevens, Jack Martin, James Pollard, Lindsay Cummings, Louise Laleye, Martha Batting, Michelle Moynihan, Monika Tomizcek, Ryan Kelsall, Sam Collins, and Tamsin Hughes. Royal Liverpool NHS Trust: Lynda Gibson (PI), Caroline Morrish, Catherine Heaton, and Kerry Mclean. Salisbury NHS Foundation Trust: Alison Wright (PI), Claire Fry, Claire Paling, Craig Evans, Karen Jocelyn, and Kate Josling. Sandwell & West Birmingham NHS Trust: Felipe Forte (PI), Anju Bhopal, Beena Borhara, Emma Fletcher, Hannah Brown, Leah O’Toole, Natasha Pathak, Sarah Calloway, and Yogita Stokes. The Royal Orthopaedic Hospital NHS Foundation Trust: Adrian Gardener (PI), Claudette Jones, Dave Rogers, Gareth Stephens, Jodie Walters, Kate MacFarlane, Lucie Gosling, Natalie Jackson, Sarah Rich, and Sue Thomas. Wirral University Teaching Hospital NHS Trust: Carole Burton (PI), Zara Hansen (PI), Clare Greenwood, Dan Hopwood, Gareth Hunt, Helen Westcott, Lynne Hardy, Robbie Ryan, and Stewart Langford. This study has been conducted as part of the portfolio of trials in the registered UKCRC Oxford Clinical Trials Research Unit (OCTRU) at the University of Oxford. It has followed their Standard Operating Procedures ensuring compliance with the principles of Good Clinical Practice and the Declaration of Helsinki and any applicable regulatory requirements.

## References

[1] de Schepper EI , Overdevest GM, Suri P, et al. Diagnosis of lumbar spinal stenosis: an updated systematic review of the accuracy of diagnostic tests. Spine (Phila Pa 1976). 2013;38(8):E469–E481. doi:10.1097/BRS.0b013e31828935ac 23385136

[2] Suri P , Rainville J, Kalichman L, Katz JN. Does this older adult with lower extremity pain have the clinical syndrome of lumbar spinal stenosis? JAMA. 2010;304(23):2628–2636. doi:10.1001/jama.2010.1833 21156951 PMC3260477

[3] Williamson E , Sanchez Santos MT, Morris A, et al. The prevalence of back and leg pain and the cross-sectional association with adverse health outcomes in community dwelling older adults in England. Spine (Phila Pa 1976). 2021;46(1):54–61. doi:10.1097/BRS.0000000000003719 33315364

[4] Ishimoto Y , Yoshimura N, Muraki S, et al. Prevalence of symptomatic lumbar spinal stenosis and its association with physical performance in a population-based cohort in Japan: the Wakayama Spine Study. Osteoarthr Cartil. 2012;20(10):1103–1108. doi:10.1016/j.joca.2012.06.018 22796511

[5] Battié MC , Jones CA, Schopflocher DP, Hu RW. Health-related quality of life and comorbidities associated with lumbar spinal stenosis. Spine J. 2012;12(3):189–195. doi:10.1016/j.spinee.2011.11.009 22193054

[6] Kreiner DS , Shaffer WO, Baisden J, et al. Evidence-based clinical guidelines for multidisciplinary spine care: diagnosis and treatment of degenerative lumbar spinal stenosis. N Am Spine Soc J. 2011;13(7):P734–P743. doi:10.1016/j.spinee.2012.11.059 23830297

[7] Rousing R , Jensen RK, Fruensgaard S, et al. Danish national clinical guidelines for surgical and nonsurgical treatment of patients with lumbar spinal stenosis. Eur Spine J. 2019;28(6):1386–1396. doi:10.1007/s00586-019-05987-2 31098717

[8] Lamb S , Hansen Z, Lall R, et al. Group cognitive behavioural treatment for low-back pain in primary care: a randomised controlled trial and cost-effectiveness analysis. Lancet. 2010;375(9718):916–923. doi:10.1016/s0140-6736(09)62164-4 20189241

[9] Williamson E , Ward L, Vadher K, et al. Better Outcomes for Older people with Spinal Trouble (BOOST) Trial: a randomised controlled trial of a combined physical and psychological intervention for older adults with neurogenic claudication, a protocol. BMJ Open. 2018;8(10):e022205. doi:10.1136/bmjopen-2018-022205 PMC6196848 30341124

[10] Marian IR , Williamson E, Garrett A, Lamb SE, Dutton SJ. Better Outcomes for Older people with Spinal Trouble (BOOST) trial: statistical analysis plan for a randomised controlled trial of a combined physical and psychological intervention for older adults with neurogenic claudication. Trials. 2020;21(1):667. doi:10.1186/s13063-020-04590-x 32693842 PMC7372766

[11] Ward L , Williamson E, Hansen Z, et al. Development and delivery of the BOOST (Better Outcomes for Older adults with Spinal Trouble) intervention for older adults with neurogenic claudication. Physiotherapy. 2019;105(2):262–274. doi:10.1016/j.physio.2019.01.019 30935673

[12] Sanchez Santos MT , Williamson E, Bruce J, et al. Cohort profile: Oxford Pain, Activity and Lifestyle (OPAL) Study, a prospective cohort study of older adults in England. BMJ Open. 2020;10(9):e037516. doi:10.1136/bmjopen-2020-037516 PMC7473632 32883729

[13] Sheehan B . Assessment scales in dementia. Ther Adv Neurol Disord. 2012;5(6):349–358. doi:10.1177/1756285612455733 23139705 PMC3487532

[14] Buckley JP , Borg GA. Borg’s scales in strength training; from theory to practice in young and older adults. Appl Physiol Nutr Metab. 2011;36(5):682–692. doi:10.1139/h11-078 21977913

[15] Chaudhry S , Jin L, Meltzer D. Use of a self-report-generated Charlson Comorbidity Index for predicting mortality. Med Care. 2005;43:607–615. doi:10.1097/01.mlr.0000163658.65008.ec 15908856

[16] Johnston MC , Crilly M, Black C, Prescott GJ, Mercer SW. Defining and measuring multimorbidity: a systematic review of systematic reviews. Eur J Public Health. 2019;29(1):182–189. doi:10.1093/eurpub/cky098 29878097

[17] Kuorinka I , Jonsson B, Kilbom A, et al. Standardised Nordic questionnaires for the analysis of musculoskeletal symptoms. Appl Ergon. 1987;18(3):233–237. doi:10.1016/0003-6870(87)90010-x 15676628

[18] Syddall HE , Westbury LD, Cooper C, Sayer AA. Self-reported walking speed: a useful marker of physical performance among community-dwelling older people? J Am Med Dir Assoc. 2015;16(4):323–328. doi:10.1016/j.jamda.2014.11.004 25523286 PMC6600869

[19] Hill JC , Dunn KM, Lewis M, et al. A primary care back pain screening tool: identifying patient subgroups for initial treatment. Arthritis Rheum. 2008;59(5):632–641. doi:10.1002/art.23563 18438893

[20] Resnick B , Jenkins LS. Testing the reliability and validity of the self-efficacy for exercise scale. Nurs Res. 2000;49(3):154–159. doi:10.1097/00006199-200005000-00007 10882320

[21] Newell AM , VanSwearingen JM, Hile E, Brach JS. The modified gait efficacy scale: establishing the psychometric properties in older adults. Phys Ther. 2012;92(2):318–328. doi:10.2522/ptj.20110053 22074940 PMC3269773

[22] Waddell G , Newton M, Henderson I, Somerville D, Main CJ. A Fear-Avoidance Beliefs Questionnaire (FABQ) and the role of fear-avoidance beliefs in chronic low back pain and disability. Pain. 1993;52(2):157–168. doi:10.1016/0304-3959(93)90127-B 8455963

[23] Laidlaw K , Power MJ, Schmidt S. The Attitudes to Ageing Questionnaire (AAQ): development and psychometric properties. Int J Geriatr Psychiatry. 2007;22(4):367–379. doi:10.1002/gps.1683 17051535

[24] Guralnik JM , Simonsick EM, Ferrucci L, et al. A short physical performance battery assessing lower extremity function: association with self-reported disability and prediction of mortality and nursing home admission. J Gerontol. 1994;49(2):M85–M94. doi:10.1093/geronj/49.2.m85 8126356

[25] Roberts HC , Syddall HE, Cooper C, Aihie Sayer A. Is grip strength associated with length of stay in hospitalised older patients admitted for rehabilitation? Findings from the Southampton grip strength study. Age Ageing. 2012;41(5):641–646. doi:10.1093/ageing/afs089 22777206

[26] Shuval K , Kohl HW, 3rd, Bernstein I, et al. Sedentary behaviour and physical inactivity assessment in primary care: the Rapid Assessment Disuse Index (RADI) study. Br J Sports Med. 2014;48(3):250–255. doi:10.1136/bjsports-2013-092901 24144532 PMC4226341

[27] Lamb SE , Jørstad-Stein EC, Hauer K, Becker C. Development of a common outcome data set for fall injury prevention trials: the Prevention of Falls Network Europe consensus. J Am Geriatr Soc. 2005;53(9):1618–16 22. doi:10.1111/j.1532-5415.2005.53455.x 16137297

[28] Gobbens RJ , van Assen MA, Luijkx KG, Wijnen-Sponselee MT, Schols JM. The Tilburg Frailty Indicator: psychometric properties. J Am Med Dir Assoc. 2010;11(5):344–355. doi:10.1016/j.jamda.2009.11.003 20511102

[29] Cleland JA , Whitman JM, Houser JL, Wainner RS, Childs JD. Psychometric properties of selected tests in patients with lumbar spinal stenosis. Spine J. 2012;12(10):921–931. doi:10.1016/j.spinee.2012.05.004 22749295

[30] Underwood MR , Barnett AG, Vickers MR. Evaluation of two time-specific back pain outcome measures. Spine. 1999;24(11):1104–1112. doi:10.1097/00007632-199906010-00010 10361660

[31] Kamper SJ , Maher CG, Mackay G. Global rating of change scales: a review of strengths and weaknesses and considerations for design. J Man Manip Ther. 2009;17(3):163–170. doi:10.1179/jmt.2009.17.3.163 20046623 PMC2762832

[32] Pua YH , Cai CC, Lim KC. Treadmill walking with body weight support is no more effective than cycling when added to an exercise program for lumbar spinal stenosis: a randomised controlled trial. Aust J Physiother. 2007;53(2):83–89. doi:10.1016/s0004-9514(07)70040-5 17535143

[33] Dunn G , Maracy M, Tomenson B. Estimating treatment effects from randomized clinical trials with noncompliance and loss to follow-up: the role of instrumental variable methods. Stat Methods Med Res. 2005;14(4):369–395. doi:10.1191/0962280205sm403oa 16178138

[34] Bohannon RW . Six-Minute walk test: a meta-analysis of data from apparently healthy elders. Top Geriatr Rehabil. 2007;23(2):155–160. doi:10.1097/01.tgr.0000270184.98402.ef

[35] Försth P , Ólafsson G, Carlsson T, et al. A randomized, controlled trial of fusion surgery for lumbar spinal stenosis. N Engl J Med. 2016;374(15):1413–1423. doi:10.1056/NEJMoa1513721 27074066

[36] Ferrucci L , Cooper R, Shardell M, Simonsick EM, Schrack JA, Kuh D. Age-related change in mobility: perspectives from life course epidemiology and geroscience. J Gerontol A Biol Sci Med Sci. 2016;71(9):1184–1194. doi:10.1093/gerona/glw043 26975983 PMC4978365

[37] Bohannon RW , Crouch R. Minimal clinically important difference for change in 6-minute walk test distance of adults with pathology: a systematic review. J Eval Clin Pract. 2017;23(2):377–381. doi:10.1111/jep.12629 27592691

[38] Leveille SG , Jones RN, Kiely DK, et al. Chronic musculoskeletal pain and the occurrence of falls in an older population. JAMA. 2009;302(20):2214–2221. doi:10.1001/jama.2009.1738 19934422 PMC2927855

[39] Sherrington C , Fairhall NJ, Wallbank GK, et al. Exercise for preventing falls in older people living in the community. Cochrane Database Syst Rev. 2019;(1):CD012424. doi:10.1002/14651858.CD012424.pub2 30703272 PMC6360922

[40] Troutman-Jordan M , Staples J. Successful aging from the viewpoint of older adults. Res Theory Nurs Pract. 2014;28(1):87–104. doi:10.1891/1541-6577.28.1.87 24772609

[41] Lyle S , Williamson E, Darton F, Griffiths F, Lamb SE. A qualitative study of older people’s experience of living with neurogenic claudication to inform the development of a physiotherapy intervention. Disabil Rehabil. 2017;39(10):1009–1017. doi:10.1080/09638288.2016.1177611 27216498

[42] Ma H , Hai B, Yan M, Liu X, Zhu B. Evaluation of effectiveness of treatment strategies for degenerative lumbar spinal stenosis: a systematic review and network meta-analysis of clinical studies. World Neurosurg. 2021;152:95–106. doi:10.1016/j.wneu.2021.06.016 34129972

[43] Wei FL , Zhou CP, Liu R, et al. Management for lumbar spinal stenosis: a network meta-analysis and systematic review. Int J Surg. 2021;85:19–28. doi:10.1016/j.ijsu.2020.11.014 33253898

[44] Anderson DB , Ferreira ML, Harris IA, et al. SUcceSS, SUrgery for Spinal Stenosis: protocol of a randomised, placebo-controlled trial. BMJ Open. 2019;9(2):e024944. doi:10.1136/bmjopen-2018-024944 PMC6398750 30765407

[45] Devereux-Fitzgerald A , Powell R, Dewhurst A, French DP. The acceptability of physical activity interventions to older adults: a systematic review and meta-synthesis. Soc Sci Med. 2016;158:14–23. doi:10.1016/j.socscimed.2016.04.006 27104307

[46] Schneider MJ , Ammendolia C, Murphy DR, et al. Comparative clinical effectiveness of nonsurgical treatment methods in patients with lumbar spinal stenosis: a randomized clinical trial. JAMA Netw Open. 2019;2(1):e186828. doi:10.1001/jamanetworkopen.2018.6828 30646197 PMC6324321

[47] Ammendolia C , Côté P, Southerst D, et al. Comprehensive nonsurgical treatment versus self-directed care to improve walking ability in lumbar spinal stenosis: a randomized trial. Arch Phys Med Rehabil. 2018;99(12):2408–2419.e2. doi:10.1016/j.apmr.2018.05.014 29935152

[48] Minetama M , Kawakami M, Teraguchi M, et al. Supervised physical therapy vs. home exercise for patients with lumbar spinal stenosis: a randomized controlled trial. Spine J. 2019;19(8):1310–1318. doi:10.1016/j.spinee.2019.04.009 30986577

[49] Minetama M , Kawakami M, Teraguchi M, et al. Supervised physical therapy versus unsupervised exercise for patients with lumbar spinal stenosis: 1-year follow-up of a randomized controlled trial. Clin Rehabil. 2021;35(7):964–975. doi:10.1177/0269215520986688 33423549

[50] Jensen RK , Harhangi BS, Huygen F, Koes B. Lumbar spinal stenosis. BMJ. 2021;373:n1581. doi:10.1136/bmj.n1581 34187838

[51] Deyo RA , Mirza SK, Martin BI, Kreuter W, Goodman DC, Jarvik JG. Trends, major medical complications, and charges associated with surgery for lumbar spinal stenosis in older adults. JAMA. 2010;303(13):1259–1265. doi:10.1001/jama.2010.338 20371784 PMC2885954

[52] Zaina F , Tomkins-Lane C, Carragee E, Negrini S. Surgical versus non-surgical treatment for lumbar spinal stenosis. Cochrane Database Syst Rev. 2016;( 1):Cd010264. doi:10.1002/14651858.cd010264.pub2 26824399 PMC6669253

[53] Torgerson DJ . Contamination in trials: is cluster randomisation the answer? BMJ (Clinical Researched). 2001;322(7282):355–357. doi:10.1136/bmj.322.7282.355 PMC1119583 11159665

[54] Hurst C , Robinson SM, Witham MD, et al. Resistance exercise as a treatment for sarcopenia: prescription and delivery. Age Ageing. 2022;51(2). doi:10.1093/ageing/afac003 PMC8840798 35150587

[55] LaFave SE , Granbom M, Cudjoe TKM, Gottsch A, Shorb G, Szanton SL. Attention control group activities and perceived benefit in a trial of a behavioral intervention for older adults. Res Nurs Health. 2019;42(6):476–482. doi:10.1002/nur.21992 31647125 PMC6858509

[56] Nicolson PJA , Bennell KL, Dobson FL, Van Ginckel A, Holden MA, Hinman RS. Interventions to increase adherence to therapeutic exercise in older adults with low back pain and/or hip/knee osteoarthritis: a systematic review and meta-analysis. Br J Sports Med. 2017;51(10):791–799. doi:10.1136/bjsports-2016-096458 28087567

[57] Comer C , Lee H, Williamson E, Lamb S. Understanding the mechanisms of a combined physical and psychological intervention for people with neurogenic claudication: protocol for a causal mediation analysis of the BOOST trial. BMJ Open. 2020;10(9):e037121. doi:10.1136/bmjopen-2020-037121 PMC7470505 32878759

