# Supplementary material for: The Clinical Effectiveness of a Physiotherapy Delivered Physical and Psychological Group Intervention for Older Adults With Neurogenic Claudication: The BOOST Randomized Controlled Trial
Source: J Gerontol A Biol Sci Med Sci. 2022 Mar 12;77(8):1654–64. doi: 10.1093/gerona/glac063 (PMC9373932; doi:10.1093/gerona/glac063)
Supplement: glac063_suppl_Supplementary_Material [file glac063_suppl_supplementary_material.pdf]

Supplementary information

Table S1 Intervention delivery fidelity checklist

Criteria	Fully achieved	Partially achieved	Not achieved	Problems identified
**BOOST Individual PT assessment**				
1. Red flag screening				
2. Medical history and medication use				
3. Current exercise and activities				
4. Neurological assessment				
5. Lumbar/ROM assessment				
6. Assessment/baseline setting for walking circuit				
7. Demonstration/baseline setting for circuit exercises				
8. Provided physiotherapy information leaflet				
9. Demonstrated flexion exercises				
10. Participant given opportunity to ask questions				
**BOOST GROUP SESSION**				
**Education/discussion session**				
1. Seating appropriately arranged				
2. All resources provided				
3. Encouraged group participation				
4. Listened appropriately				
5. Empathy demonstrated				
6. Elicited beliefs/thoughts				
7. Questioning style demonstrated				
8. Appropriate pacing of session				
9. Facilitated participant problem solving				
10. Presented crib sheet content				
11. Education session completed within time limit				
**Exercise circuit**				
12. Warm-ups conducted per protocol				
13. Strengthening exercises conducted per protocol				
14. Balance exercises conducted per protocol				
15. Flexibility exercise conducted per protocol				
16. Circuit stations well set-up (include rest stations)				
17. Exercises recorded on attendance and exercise log for each participant				
18. Home exercise planners discussed and completed for each participant (from session 5 only)				

Criteria	Fully achieved	Partially achieved	Not achieved	Problems identified
19. Exercise session completed within time limit				
20. No off-protocol exercises delivered				
**Walking circuit**				
21. Walking circuit stations well set-up (lighting, obstacles, rest stations)				
22. Appropriate use of weights (hand weights only)				
23. Adaptations provided as appropriate e.g challenging obstacles; lap counters				
24. Appropriate progression of walking				
25. Discussed home practice of walking				
26. Walking recorded on attendance and exercise log for each participant				
27. Home walking planners discussed and completed for each participant (from session 8 only)				
28. Walking session completed within time limit				
29. No off-protocol exercises delivered				
**BEST PRACTICE ADVICE**				
**Assessment**				
1. Red flag screening				
2. Medical history/medication use				
3. Current mobility/walking aid use				
4. Current exercise & activities				
5. Neurological assessment				
6. Lumbar ROM				
7. Walking assessment				
**Education and advice**				
8. Education about spinal stenosis and neurogenic claudication				
9. Pain mechanisms				
10. Exercises demonstrated and practiced including flexion exercises				
11. Medication				
12. Walking aids				
13. Provided & explained participant information leaflet				
14. Participant given opportunity to ask questions				
15. Follow-up/Discharge advice				
16. No-off protocol content delivered				

Table S2: Adverse events

A safety reporting protocol was developed to manage the reporting of related and unexpected serious adverse events (SAEs) and directly attributable adverse events (AEs). An AE was defined as any untoward medical occurrence in a participant during a trial. Given the age range of the study population and the nature of physical interventions, we expected there to be foreseeable occurrences (serious adverse events and adverse events) that occurred during the study period. These included but not limited to acute infections (e.g. viral), medical instability (e.g. diabetic control – becomes hypoglycaemic, deterioration in control of heart failure), vestibular disorders and stroke and fall-related injuries. To capture any AEs events related to the interventions, if any of these occurred as the result of an incident during, or within two hours of completing the exercise sessions or follow on physical activities, or were related to the intervention, they were then categorised as a serious adverse events or adverse event and the safety reporting protocol was followed.

Physiotherapists delivering the interventions and all research staff were trained in identifying and reporting AEs. Reporting was done via standardised reporting template. AEs were primarily identified during intervention sessions by the physiotherapists, at follow up research clinics by research staff or when research staff contacted participants by telephone to collect core outcomes. This may have resulted in under reporting from participants in the BPA arm and more reports from participants allocated to the BOOST programme. BOOST programme participants had more contact with the physiotherapists and therefore more opportunity to report adverse events. However, due to the nature of the interventions, we were more concerned about AEs related to the more intensive and newer intervention (BOOST programme) and our safety reporting protocol ensured that we collected these.

BOOST Programme (n=292)				BPA (n=143)		
**Serious Adverse events**		1 (<1%)			**0**	
	Cardiac symptoms requiring investigation	1	Not related			
**Adverse events**		12 (4.1%)			2 (1.4%)	
	Aggravation of joint pain (3 knee pain, 1 back pain, 1 back and knee pain)	5	2 definitely related, 3 possibly related	Abdominal hernia (no treatment needed)	1	Unlikely to be related
	Increased neurogenic claudication symptoms	2	2 probably related	Joint pain (elbow and knee)	1	Probably related
	Became very fatigued during walking circuit	1	Probably related			
	Fall during walking circuit – no injuries reported	1	Definitely related			
	Pain in shin - muscular or pressure from weights	1	Possibly related			
	Skin irritation by ankle weights used at home	1	Definitely related			
	Panic Attack during baseline assessment.	1	Possibly related			

Table S3 Participant satisfaction

	Best Practice Advice		BOOST Programme	
	**n**	**%**	**n**	**%**
**Satisfaction with treatment**				
6 months				
(Very dissatisfied) 0	8	6%	11	4%
1	2	2%	10	4%
(Neither satisfied nor dissatisfied) 2	47	38%	51	20%
3	28	22%	62	24%
(Very satisfied) 4	40	32%	122	48%
12 months				
(Very dissatisfied) 0	7	6%	12	5%
1	9	7%	5	2%
(Neither satisfied nor dissatisfied) 2	49	39%	62	25%
3	28	22%	66	27%
(Very satisfied) 4	33	26%	103	42%
**Satisfaction with change in back and leg problems**				
6 months				
(Very dissatisfied) 0	11	9%	12	5%
1	12	10%	17	7%
(Neither satisfied nor dissatisfied) 2	60	48%	95	37%
3	26	21%	65	25%
(Very satisfied) 4	16	13%	67	26%
12 months				
(Very dissatisfied) 0	17	13%	23	9%
1	5	4%	9	4%
(Neither satisfied nor dissatisfied) 2	57	45%	97	39%
3	24	19%	59	24%
(Very satisfied) 4	23	18%	59	24%
